# The European Violence in Psychiatry Research Group (EViPRG): what does it offer for a European psychiatrist?

**DOI:** 10.1192/j.eurpsy.2025.282

**Published:** 2025-08-26

**Authors:** T. Lantta, S. Hirsch, T. Hatling

**Affiliations:** 1 Department of Nursing Science, University of Turku, Turku, Finland; 2 Centre for Forensic Behavioural Science, Swinburne University of Technology, Melbourne, Australia; 3 Department of Biomedical, Metabolic and Neuroscience, University of Modena and Reggio Emilia, Reggio Emilia, Italy; 4 ZfP Suedwuerttemberg, Ravensburg; 5 Ulm University, Ulm, Germany; 6 Norwegian resource center on community mental health, N/A, Norway

## Abstract

**Introduction:**

The use of restrictive practices such as restraint, seclusion and long-term segregation on people with mental health problems remains common in European psychiatric care to manage patients’ violent and other challenging behaviour. These practices violate human rights and thus there is a growing international policy move to reduce or even ultimately stop using them. To achieve this, clinicians, researchers, teachers, trainers, policy-makers and user representatives need to collaborate to transform psychiatric services towards non-coercive services. An international network provides one way towards this vision.

**Objectives:**

Here we present an international network focusing on developing knowledge and practices aiming at reducing violence and coercion in mental health settings. We will illustrate how networking in an interdisciplinary group can be beneficial to both European psychiatrists as well as other professionals in mental health services.

**Methods:**

The EViPRG is a non-governmental research-focused network founded in 1997. Our vision is to work together to improve competency and quality of practice with the aim to reduce coercion and violence in mental health services, and address ongoing human rights issues. Clinicians in the psychiatric field as well as researchers can join the network through an electronic application (https://www.eviprg.eu/). Participation of early career researchers is encouraged.

**Results:**

The EViPRG offers a unique network to connect with like-minded colleagues, collaborate on research projects, learn from various national initiatives to reduce coercion, exchange best practice models and take part in discussions via our various platforms. The network meets 3-4 times per year both in-person and online. Meetings provide an arena to present the latest research findings, generate new research projects and get feedback from colleagues. The EViPRG also organises the bi-annual “European Conference on Violence in Clinical Psychiatry” and members get a reduced fee to attend.

**Conclusions:**

The network has more than 130 members in Europe and beyond. Numerous multi-country studies have been initiated through the network. As an example, in the years 2021-2024, a European Commission-funded project COST Action FOSTREN widened the network to new countries. As a result, we expect a rise in our membership, especially from Eastern Europe. If you want to find like-minded research partners and innovators, link in with a strong community aspiring to influence policy and practice in this area, progress your career and international profile, or just meet new colleagues, membership in the EViPRG can be your choice.

**Disclosure of Interest:**

None Declared

